# History of a Case of Traumatic Cataract

**Published:** 1850-05

**Authors:** C. Green

**Affiliations:** Homer, New York


ART. II.—*History of a case of Trumatic Cataract.* By C. GREEN, M. D., Homer, New York.

The following case was treated conjointly by my friend Dr. P. H. Burdick, of Preble, and myself, and through his courtesy I am permitted to report it.

May 12, 1848, Mrs. M. H., of Preble, aged 29 was removing carpet tacks, when one was projected with considerable force into the right eye, piercing the cornea on its inner aspect, and, as the sequel shows, crossed the anterior chamber, entered the pupil and wounded the lens posterior to the external edge of the pupil. She seized the head of the nail which was found resting in the internal canthus, and extracted it. The extraction was followed by a stream of aqueous humor. She fainted, although the pain was not severe. The sight immediately became dim, and has continued so to the present time.

May 20th, she consulted me at the suggestion of Dr. Burdick, who first saw the case. Soon after the accident, the eye became very sensible to light, insomuch that she was obliged to confine herself to a dark room. The present state of the eye is as follows:—There is some conjunctival inflammation, and still there is but little pain, The cornea is perfectly clear, with the exception of a scarcely perceptible cicatrix where the tack entered; the iris natural in color; the anterior segment of the capsule of the crystalline lens is lacerated, and a portion of the external soft part of the crystalline humor is protruding through the opening in the capsule, across the posterior chamber, through the pupil, into the anterior chamber and, when passing the pupil, rests on its external edge, and pushes forward the iris at that point. This protruding portion of the lens has a semi-opaque jelly-like appearance of a rounded form, and occupies a little more than a third of the lower and external portion of the pupil. The laceration of the capsule is primarily behind the external segment of the iris. The unlacerated portion of the anterior capsule is semi-opaque. Her husband informs me, that immediately after the accident, he saw, just at the external border of the pupil, the same appearance which is now presented, but not to so great an extent,—that this protruded substance began to thin down and finally disappeared on the 4th day, leaving a hazy appearance of the pnpil. Yesterday a fresh portion of the lens made its appearance, and the part now projecting into the anterior chamber is three-forths of a line in width and length. For two or three days she has been able to bear the light much better—even to go out with the eye unprotected. She can discern with this eye the outlines of objects—even the large plaid of her dress.

I directed the observance of absolute rest in a darkened room, laxatives, and the application of a solution of Nit. argt. to the conjunctiva.*

May 26th, the patient was again in town. The sol. nit. argt. had been applied the day after I last saw her. The pain increased. It was a deep-

* I should omit the latter application in a similar case again, for fear of exciting too great activity of the ciliary vessels, and causing internal inflammation.

seated, "bursting" pain. She reduced the solution one-half, but the eye still continued painful. Yesterday omitted the solution, and the pain in some degree subsided. To-day rode 8 miles to town—bright sun-light—eye quite painful after arrival. Applied a leech near the external canthus, and promoted the bleeding for an hour and a half. This gave more sensible relief than she had before experienced since the accident. Directed that a solution of the extract of belladonna be dropped into the eye, if she felt much of the deep-seated pain. Recommended the further employment of leeches, if the conjunctival inflammation and vascular turgescence continue. I should have stated that the protruding portion of the lens has increased, and now fills two-thirds of the pupil—capsule a little more opaque—can bear light a little better.

June 9th, saw the patient at her house. The belladonna when, first used, seemed to give the eye great relief, but afterwards caused pain, as did all applications;—had had severe paroxysms of pain, and had kept more or less under the influence of morphine. Had taken blue mass every other night. The extruded portion of the crystalline humor less regular in its outlines, and more opaque. The inflammatory symptoms were increased, and there were marked evidences of the existence of iritis. In consultation with Dr. Burdick, determined to mercurialize her promptly. R. ext. belladonna gr. 1; bi-chloride of mercury gr 1-12th these times a day.

June 22. The conjunctival inflammation is less. The iris appears more vascular—pupil contracted—protruding portion of the lens diminished four-fifths; capsule clearer; the pain throbbing, but not so severe as at the last date; has some pain in the other eye, with apparently a little vascularity of the iris. She is weak, but the countenance looks better than before. Owing to circumstances, over which neither Dr. Burdick nor I had any control, the last prescription was only partially fulfilled, and hence mercurial action had not been induced. Advised rapid salivation, dilatation of the pupils with belladonna, and the application of blisters to the mastoid or nucha.

July 7th. The patient continued the mercurial from the last date for four or five days, when it produced its constitutional effects. She at the same time (June 23d) applied a blister to the neck which produced so much morbid sensibility as to increase the pain in the eye, but which subsided as soon as the blister had fully drawn, and began to heal. The iritis began to subside as soon as the general effects of the mercury were observed. The blister also appeared to be productive of benefit. Has taken ext. belladonna at night to the extent of producing dilatation of the pupil.

The albuginea is of a dull white color; a very dim pink zone surrounded the cornea; iris rather duller than natural; pupil contracted and a portion of it attached to the lens by a band of organized fibrin. The protruding portion of the lens is dissolved down to a level with the capsule, and is of a brownish or dirty gray color, surrounded by the white edge of the lacerated capsule. She bears light much better, and thinks the power of vision is improving. Can see better when the object is on the right side of her, for the reason that the reflected rays then reach the retina through the less injured portion of the capsule, the opacity of which is now scarcely perceptible. Her general health is better. Advised the use of belladonna to be applied to the lids to produce dilation and break up the adhesion.

July 25th. The patient bears light very well. Can see better in twilight than in full sunlight, and by lamp light when it is behind her.

Aug. 14th. Iris dull; pupil contracted—expands but slightly in twilight and by light of the lamp. A portion of the anterior capsule appears to be perfectly transparent. Portion of the pupilary margin of the iris still bound to the lens by organized fibrin. Had not used the belladonna as I directed on the 7th ult. Still urged its application.

Aug 29th. Patient had this morning applied the moistened extract to the upper lid, which produced unequal dilation of the pupil—the upper sixth of the pupil dilating, and the rest remaining in *statu quo*. The upper portion of the lenticular capsule (which was the only portion which the fixed state of the pupil would allow me to obsere,) was quite opaque. Advised further use of the belladonna.

Dec. 25th. Saw the patient in town to-day. She had used the belladonna from time to time, with the effect, only, mentioned at the last date, until about two weeks ago, when she applied to the lids a belladonna plaster, at night, and on the following morning found that the whole of the adhesion of the edge of the pupil to the lens had broken up—with the exception, perhaps, of a small point on the lower margin. She can now see well enough to recognize a person, if the light falls fully upon him. Can see better in the strong sunlight than in twilight. There appears to be a small space in the centre of the lens, which is transparent—perhaps, half a line in length, and one-fourth of a line in width. Advised the application of ext. bell. once a week for four or five weeks, for the purpose of preventing new adhesions. No pain or inflammation was produced by the breaking up of the adhesions.

Jan 20th, 1849.—The transparent space in the anterior capsule has increased one-half.

Feb. 14.—The patient has been imprudently indulging in domestic labor. The transparent portion of the capsule is diminishing; the opacity of the rest of the capsule more dense. There is more or less pain in the eye, especially on exercising much near a fire. Directed the use of laxatives, tartar emetic ointment behind the ear to pustulation, and ext. bell. to the lids.

May 28. The last prescription had a favorable effect. The pain began to subside, and the opacity to disappear, as soon as the pustules began to form, and at this time the capsule is transparent in the centre to the extent of a line in length, and half a line in width—the length being transverse. The lower third of the pupilary margin of the iris adheres to the lens. The pupil does not fully dilate; can read the heading of a newspaper, and recognize a person six yards off. The eye is myopic and weak.

Feb., 1850.—The eye now presents nearly the same appearance noticed at the last date. There is a greyish white opacity of the anterior segment of the capsule encircling the transparent space in the centre. The pupil is smaller, and not as mobile as its fellow,—a consequence, doubtless, of the organic alteration of the tissues of the iris by inflammation. The power of vision exists in about the same degree as at the last date, but it is occasionally diminished by the causes which tend to excite pain and inflammation. At times, she suffered considerable pain in the eye, especially after fatiguing exercises over a cooking stove. The other eye sympathizes strongly with the wounded one, and suffers with it from morbid sensibility—" weakness."

*Remarks*.—In this case the lens did not become opaque, as is frequent in as extensive lacerations as occurred in this instance, but, with the exception of the locality of the wound, remained transparent, and was, therefore, not absorbed; for it is in accordance with the observation of ophthalmic surgeons, that so long as the lens maintains its transparency, it will not be absorbed. It has been observed by various surgeons, to continue transparent ever after being deprived of its capsule. The wound in the capsule, instead of enlarging, in this case, diminished. Opportunity has not offered, for the last year, to examine this eye catoptrically, and with the pupil extensively dilated, and I much regret this imperfection in the history.

It will be of practical utility to observe one fact in the progress of this case, namely, the facility with which of the usually transparent portion of the capsule could be rendered opaque by over exercise—especially by that kind of exercise which much disturbed the circulation of the head, as working over a hot stove. The observance of rest, and the use of antiphlogistics were of marked efficacy. This was to be expected in a case of traumatic cataract, and I may add, *should* be expected in many cases of spontaneous cataract, notwithstanding the fact that some members of the profession have sneered at the pathology and treatment of such cases, as laid down by Prof. Bryan* and a few others. I am fully impressed with the conviction that many a lens and its capsule which now are permitted to become permanently opaque, might be saved from this condition by a little careful observation, and a due regard to a few well settled general principles.

HOMER, March, 1850.

*See Buffalo Med. Jour., October, 1848, p. 367.

